# Erythropoietin Levels in Elderly Patients with Anemia of Unknown Etiology

**DOI:** 10.1371/journal.pone.0157279

**Published:** 2016-06-16

**Authors:** Zachary Gowanlock, Swetha Sriram, Alison Martin, Anargyros Xenocostas, Alejandro Lazo-Langner

**Affiliations:** 1 Department of Medicine, Division of Hematology, Western University, London, ON, Canada; 2 Department of Oncology, Western University, London, ON, Canada; 3 Department of Epidemiology and Biostatistics, Western University, London, ON, Canada; University of Luebeck, GERMANY

## Abstract

**Background:**

In many elderly patients with anemia, a specific cause cannot be identified. This study investigates whether erythropoietin levels are inappropriately low in these cases of “anemia of unknown etiology” and whether this trend persists after accounting for confounders.

**Methods:**

This study includes all anemic patients over 60 years old who had erythropoietin measured between 2005 and 2013 at a single center. Three independent reviewers used defined criteria to assign each patient’s anemia to one of ten etiologies: chronic kidney disease, iron deficiency, chronic disease, confirmed myelodysplastic syndrome (MDS), suspected MDS, vitamin B_12_ deficiency, folate deficiency, anemia of unknown etiology, other etiology, or multifactorial etiology. Iron deficiency anemia served as the comparison group in all analyses. We used linear regression to model the relationship between erythropoietin and the presence of each etiology, sequentially adding terms to the model to account for the hemoglobin concentration, estimated glomerular filtration rate (eGFR) and Charlson Comorbidity Index.

**Results:**

A total of 570 patients met the inclusion criteria. Linear regression analysis showed that erythropoietin levels in chronic kidney disease, anemia of chronic disease and anemia of unknown etiology were lower by 48%, 46% and 27%, respectively, compared to iron deficiency anemia even after adjusting for hemoglobin, eGFR and comorbidities.

**Conclusions:**

We have shown that erythropoietin levels are inappropriately low in anemia of unknown etiology, even after adjusting for confounders. This suggests that decreased erythropoietin production may play a key role in the pathogenesis of anemia of unknown etiology.

## Introduction

Anemia is a significant and frequently overlooked problem in elderly patients and is associated with increased mortality, frailty and reduced quality of life.[1–4] In contrast to younger populations, the etiology of anemia in the elderly is often unclear and the term “anemia of unknown etiology” (AUE) is used when investigations do not suggest a specific cause. AUE accounts for 37 to 45 percent of the cases of anemia in the elderly.[5–7]

Erythropoietin (EPO) is a hormone that plays an important role in the regulation of erythropoiesis and is mainly released from the kidneys in response to tissue hypoxia. The primary action of EPO is to promote the proliferation and differentiation of the colony-forming unit-erythroid (CFU-E) and other erythroid progenitors.[8] It has been suggested that elderly patients with AUE may have inadequate EPO responses to anemia.[9] A previous meta-analysis by our group[10] has found that the endogenous EPO levels in AUE seem to be significantly lower than in iron deficiency anemia (IDA) and anemia of chronic disease (ACD), yet are slightly higher than the EPO levels in anemia of chronic kidney disease (CKD), suggesting that EPO levels may be inappropriately low in AUE. However, previous studies have been limited by methodological considerations, most importantly by the possibility of residual confounding from unaccounted variables. Hence, this study aimed to investigate the patterns of EPO response in elderly patients with AUE in comparison to other clinically determined etiologies in a large cohort, while accounting for potentially relevant confounders.

## Methods

### Study Design and Patient Population

We conducted a retrospective cohort study including all consecutive patients referred to the Division of Hematology at the London Health Sciences Centre, a university-affiliated academic centre in London, Ontario, Canada, and who had erythropoietin (EPO) levels determined between January 1, 2005 and December 31, 2013. Patients were identified using laboratory records and EPO levels were determined by chemiluminescence using an immunoenzymatic method (Access EPO Erythropoietin) using an Access 2 Analyzer (Beckman Coulter CA, USA) with a reference range of 2.59 to 18.50 IU/L. We included patients 60 years or older who met the World Health Organization criteria for anemia (<130 g/L in men, <120 g/L in women)[11] excluding patients with insufficient electronic medical records. The study was approved by the Health Sciences Research Ethics Board (REB) at Western University. Written patient consent was not requested, and patient information was anonymized and de-identified prior to analysis.

### Determination of Anemia Etiology

The etiology of each patient’s anemia was adjudicated to one of ten diagnostic groups: chronic kidney disease (CKD), iron deficiency anemia (IDA), anemia of chronic disease (ACD), confirmed myelodysplastic syndrome (MDS), suspected MDS, vitamin B12 deficiency, folate deficiency, anemia of unknown etiology (AUE), other miscellaneous etiology, or multifactorial. Three investigators blinded to EPO levels independently reviewed each patient’s electronic medical records and assigned an etiology based on defined criteria (Table 1). The etiology reported by at least 2 of the 3 reviewers was used in the analysis, with differences resolved by consensus after consultation with a fourth reviewer.

**Table 1:** Criteria used to assign an etiology to a patient’s anemia.

Anemia etiology	Definition
Chronic kidney disease	The estimated glomerular filtration rate estimated by the CKD-EPI formula is less than 30 mL/min/1.73 m^2^ and chronic kidney disease is considered to be the primary cause of the anemia.
Iron deficiency	The serum ferritin was less than 50 ng/mL or the bone marrow shows absent iron stores, and iron deficiency is considered to be the primary cause of the anemia.
Chronic disease	The patient had a diagnosis of a chronic inflammatory disorder that was considered to be the primary cause of the anemia. Chronic inflammatory disorders include vasculitis, connective tissue diseases, autoimmune diseases, rheumatoid arthritis, polymyalgia rheumatica, giant cell arteritis and inflammatory bowel disease.
Myelodysplastic syndrome (confirmed)	The bone marrow biopsy, aspirate or cytogenetic workup was considered to be diagnostic of myelodysplastic syndrome.
Myelodysplastic syndrome (suspected)	Myelodysplastic syndrome was considered to be highly probable based on clinical and laboratory features, but the bone marrow was not sampled to confirm the diagnosis.
Vitamin B12 deficiency	The serum vitamin B12 concentration was less than 148 pmol/L and vitamin B12 deficiency was considered to be the primary cause of the anemia.
Folate deficiency	The erythrocyte folate concentration was less than 340 nmol/L and folate deficiency was considered to be the primary cause of the anemia.
Anemia of unknown etiology	An etiology of the anemia cannot be identified based on the clinical and laboratory investigations.
Other etiology	The etiology of the anemia was identified but does not fall into the preceding eight categories.
Multifactorial etiology	The criteria for multiple categories are met and there was no clear indication as to which etiology was the primary cause of the anemia.

### Data Collection

All data was obtained from the electronic medical records. For patients with multiple EPO determinations only the first measurement was used in the analysis. Information on age, comorbidities and laboratory values were collected at the time the EPO level was measured.

The CKD-EPI[12] formula was used to estimate the glomerular filtration rate in each patient since it was shown to more accurately classify future risk of end-stage renal disease and mortality compared to MDRD (Modification of Diet in Renal Disease).[13] A GFR cutoff of less than 30 mL/min/1.73 m^2^ was chosen since Mercadal et al. demonstrated that in anemic patients there is a negative correlation between GFR and hemoglobin at GFRs below 30 mL/min/1.73 m^2^ but no correlation above 30 mL/min/1.73 m^2^, suggesting that the physiologic response to anemia is somewhat preserved in patients with GFRs greater than 30 mL/min/1.73 m^2^.[14]

Serum ferritin was used in the classification criteria for iron deficiency anemia because the use of alternative indices such as the TfR-F index (the ratio of serum transferrin receptor to log ferritin)[15], transferrin saturation or CRP could not be obtained for most of the patients since out of the 570 patients, only 132 patients had iron studies (which includes transferrin and iron) and only 58 patients had available CRP values.

### Statistical Analysis

Inter-observer agreement was assessed using Kappa[16] and Fleiss' Kappa[17] statistics. A plot of EPO versus hemoglobin was constructed for each etiology group. The exponential curve of best fit was determined by the method of least squares, and the individual data points were not displayed for clarity. A reference group of patients with IDA and measured EPO levels served as an approximation for a normal EPO response to anemia. The EPO levels in each etiology group were compared with the IDA group using unpaired t-tests.

To adjust for potential confounders, we constructed stepwise linear regression models that estimated the effect of each etiology on EPO levels compared to the reference group (IDA). Models were assessed by residual analysis and EPO concentration was logarithmically transformed to maintain the assumption of homoscedasticity. Final model coefficients were back-transformed using the exponential function and thus they represent the ratio of the EPO level for each etiology relative to the reference group (i.e. a coefficient of 1 represents no difference between the EPO levels in patients assigned a specific etiology and those in the IDA group). Models were adjusted for hemoglobin level, estimated glomerular filtration rate (eGFR) using the CKD-EPI formula, and comorbidity which was determined using the Charlson’s Comorbidity Index as modified by Quan et al.[18] P-values less than 0.05 were considered to be statistically significant. Statistical analyses were completed using Excel 2007 (Microsoft Corp., Redmond WA, USA) or SPSS Statistics version 22 (IBM Corp., Armonk NY, USA).

## Results

### Patient Population

**Fig 1:**
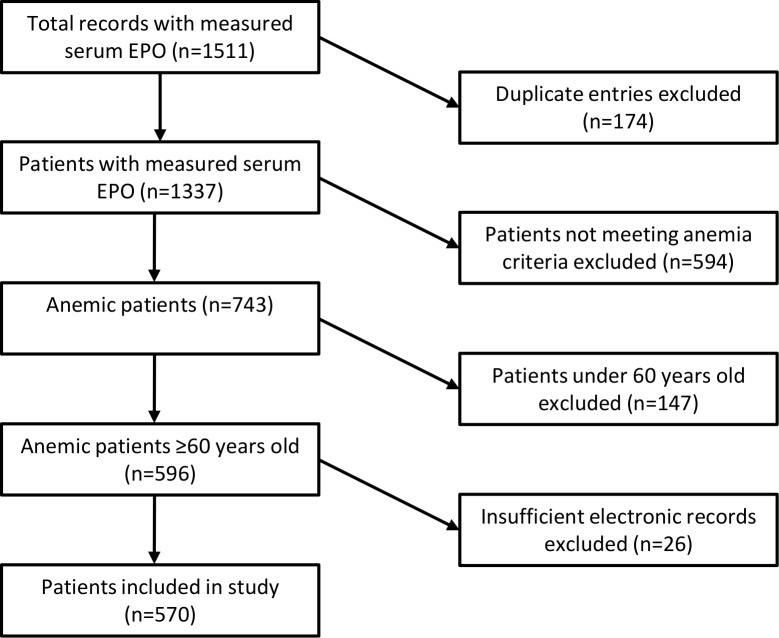
Flowchart of cohort integration. Serum EPO was measured in 1511 cases, of which 570 individual patients met inclusion criteria and were included in the study.

During the study period there were 1511 requests for serum EPO concentration measurement. We excluded 941 instances and 570 patients were ultimately included in the study. There was one case of B_12_ deficiency and no cases of folate deficiency. A flowchart of the cohort construction is shown in Fig 1.

The 3-way inter-rater agreement between the adjudicated etiologies measured by Fleiss’ Kappa was 0.591. Two-way inter observer agreements assessed by Cohen’s Kappa statistics ranged between 0.529 and 0.635. Of the 570 patients included in the study, CKD was the principal etiology in 25 patients (4.4%), IDA in 59 (10.4%), ACD in 31 (5.4%), confirmed MDS in 180 (31.2%) and AUE in 117 (20.5%). The mean age was 75.7 years and 60% were male. The mean Charlson’s Comorbidity Index was 1.5. The mean hemoglobin concentration of the entire population was 95.9 g/L. The mean erythropoietin concentration was 25.1 IU/L in CKD, 102.4 IU/L in IDA, 26.0 IU/L in ACD, 287.8 IU/L in confirmed MDS and 39.1 IU/L in AUE. Baseline clinical and laboratory characteristics for each group were collected (S1 and S2 Tables).

### Relationship between etiology and EPO level

**Fig 2:**
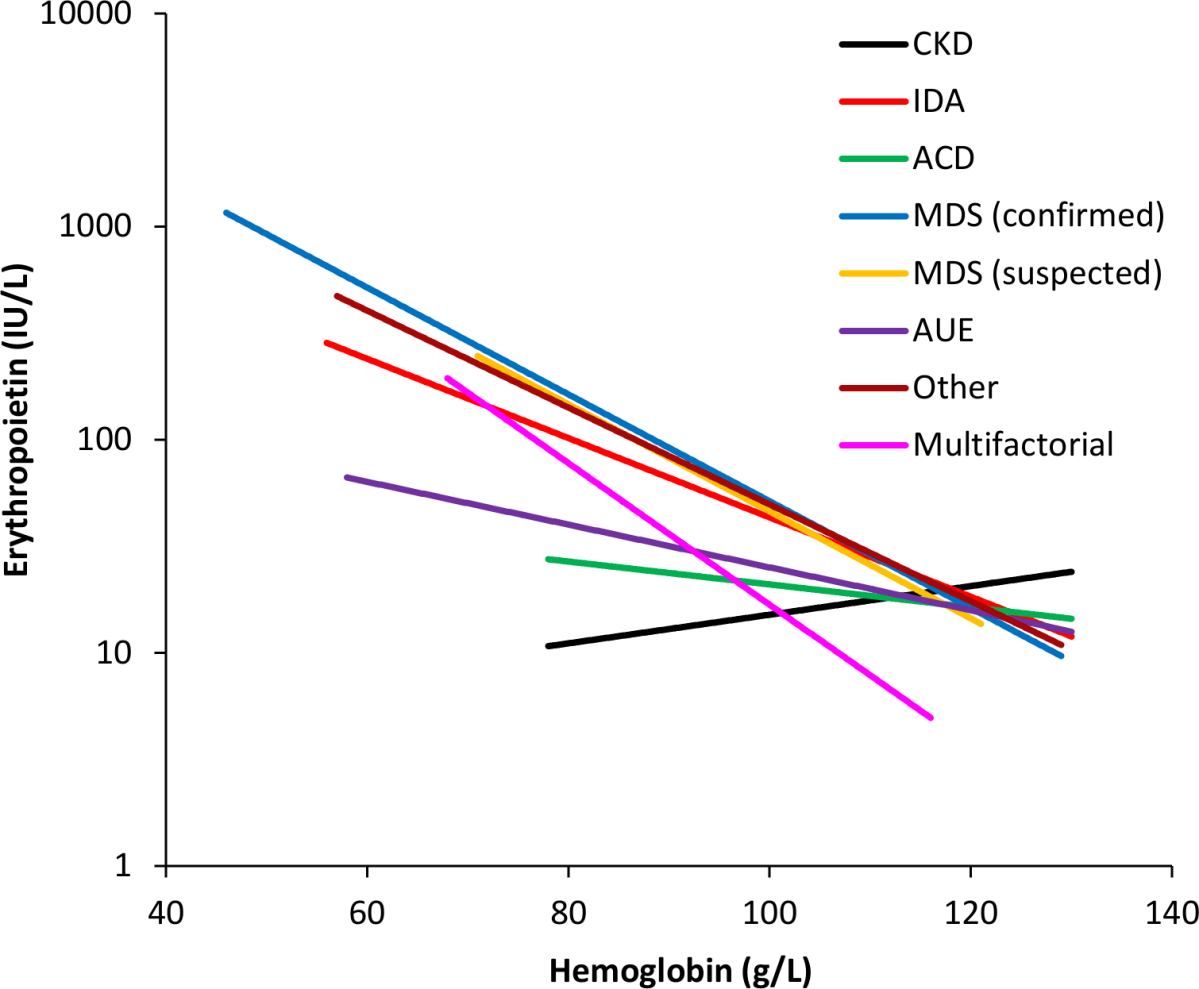
Plot of EPO versus hemoglobin. The correlation between EPO and hemoglobin for each anemia etiology is modeled by an exponential curve of best fit.

Fig 2 shows the EPO levels with respect to hemoglobin concentrations for each etiology. A comparison of the EPO level, hemoglobin and eGFR for each etiology with respect to IDA is shown (Table 2). Compared to IDA, the mean EPO levels in CKD, ACD and AUE were significantly lower whereas the EPO levels for confirmed MDS and other etiologies were higher. The mean hemoglobin level was significantly higher in AUE and lower in confirmed MDS when compared to IDA. The mean eGFR was significantly lower in the CKD, AUE and multifactorial etiology groups compared to IDA.

**Table 2:** EPO, hemoglobin and eGFR for each category of anemia etiology compared to iron deficiency anemia.

Etiology of the anemia	n	Erythropoietin, IU/L		Hemoglobin, g/L		eGFR (CKD-EPI), mL/min/1.73 m^2^	
		Mean	p-value^a^	Mean	p-value^a^	Mean	p-value^a^
Iron deficiency	59	102.4	-	95.6	-	63.3	-
Chronic kidney disease	25	25.1	0.001^b^	96.6	0.794	20.2	<0.001 ^b^
Chronic disease	31	26.0	<0.001 ^b^	99.9	0.204	57.4	0.251
MDS (confirmed)	180	287.8	0.002 ^b^	91.1	0.044 ^b^	63.7	0.900
MDS (suspected)	19	148.1	0.425	97.0	0.739	61.6	0.732
Anemia of unknown etiology	117	39.1	0.003 ^b^	105.7	<0.001 ^b^	53.6	0.008 ^b^
Other etiology	118	271.4	0.002 ^b^	92.7	0.275	62.7	0.882
Multifactorial etiology	20	106.1	0.934	93.2	0.539	37.8	<0.001 ^b^

^a^ The p-value is calculated using an unpaired t-test comparison against iron deficiency

^b^ p<0.05

Linear regression model coefficients, both unadjusted and adjusted for hemoglobin, eGFR and Charlson’s comorbidity index, were calculated (Table 3). After adjusting for hemoglobin concentration and eGFR, the EPO level was significantly lower in CKD (β coefficient, 0.52; 95% CI, 0.29–0.92, p = 0.025), ACD (β coefficient, 0.54; 95% CI, 0.37–0.77, p = 0.001) and AUE (β coefficient, 0.73; 95% CI, 0.55–0.97, p = 0.028) compared to IDA but no differences were found in the other groups. Adjustment for Charlson’s comorbidity index had very little effect.

**Table 3:** Regression coefficients for unadjusted and adjusted models of EPO levels by etiology.

**Unadjusted model**^**a**^				
**Etiology of the anemia**	**R**^**2**^_**adj**_	**Coefficient**^**e**^	**95% CI**	**p-value**
Chronic kidney disease	0.214	0.29	0.18, 0.48	< 0.001 ^f^
Chronic disease	0.137	0.42	0.27, 0.66	< 0.001 ^f^
Myelodysplastic syndrome (confirmed)	0.019	1.65	1.08, 2.51	0.020 ^f^
Myelodysplastic syndrome (suspected)	-0.010	1.04	0.54, 2.01	0.898
Myelodysplastic syndrome (all)	0.014	1.58	1.04, 2.40	0.031 ^f^
Anemia of unknown etiology	0.148	0.43	0.31, 0.58	< 0.001 ^f^
Other etiology	0.006	1.40	0.89, 2.20	0.151
Multifactorial etiology	0.031	0.54	0.29, 1.04	0.065
**Model adjusted for hemoglobin**^**b**^				
**Etiology of the anemia**	**R**^**2**^_**adj**_	**Coefficient**^**e**^	**95% CI**	**p-value**
Chronic kidney disease	0.338	0.29	0.19, 0.46	< 0.001 ^f^
Chronic disease	0.357	0.49	0.33, 0.72	< 0.001 ^f^
Myelodysplastic syndrome (confirmed)	0.324	1.30	0.91, 1.84	0.147
Myelodysplastic syndrome (suspected)	0.346	1.13	0.67, 1.92	0.638
Myelodysplastic syndrome (all)	0.332	1.28	0.91, 1.80	0.161
Anemia of unknown etiology	0.339	0.61	0.46, 0.82	0.001 ^f^
Other etiology	0.320	1.21	0.83, 1.76	0.322
Multifactorial etiology	0.386	0.48	0.29, 1.24	0.006 ^f^
**Model adjusted for hemoglobin and eGFR**^**c**^				
**Etiology of the anemia**	**R**^**2**^_**adj**_	**Coefficient**^**e**^	**95% CI**	**p-value**
Chronic kidney disease	0.389	0.52	0.29, 0.92	0.025 ^f^
Chronic disease	0.421	0.54	0.37, 0.77	0.001 ^f^
Myelodysplastic syndrome (confirmed)	0.387	1.30	0.93, 1.82	0.118
Myelodysplastic syndrome (suspected)	0.421	1.14	0.69, 1.87	0.600
Myelodysplastic syndrome (all)	0.393	1.29	0.93, 1.79	0.130
Anemia of unknown etiology	0.410	0.73	0.55, 0.97	0.028 ^f^
Other etiology	0.419	1.09	0.77, 1.56	0.626
Multifactorial etiology	0.509	0.78	0.47, 1.30	0.345
**Model adjusted for hemoglobin, eGFR and comorbidity index**^**d**^				
**Etiology of the anemia**	**R**^**2**^_**adj**_	**Coefficient**^**e**^	**95% CI**	**p-value**
Chronic kidney disease	0.405	0.49	0.28, 0.87	0.015 ^f^
Chronic disease	0.432	0.51	0.36, 0.74	0.001 ^f^
Myelodysplastic syndrome (confirmed)	0.386	1.29	0.92, 1.81	0.134
Myelodysplastic syndrome (suspected)	0.419	1.19	0.72, 1.97	0.500
Myelodysplastic syndrome (all)	0.392	1.27	0.92, 1.77	0.150
Anemia of unknown etiology	0.417	0.72	0.55, 0.96	0.025 ^f^
Other etiology	0.416	1.09	0.76, 1.55	0.636
Multifactorial etiology	0.506	0.74	0.43, 1.26	0.262

^a^ Unadjusted model: ln *EPO* = *B* ∙ *etiology*

^b^ Model adjusted for hemoglobin: ln *EPO* = *B* ∙ *etiology* + *C*_1_ ∙ *Hb*

^c^ Model adjusted for hemoglobin and eGFR: ln *EPO* = *B* ∙ *etiology* + *C*_1_ ∙ *Hb* + *C*_2_ ∙ *eGFR*

^d^ Model adjusted for hemoglobin, eGFR and comorbidity index: ln *EPO* = *B* ∙ *etiology* + *C*_1_ ∙ *Hb* + *C*_2_ ∙ *eGFR* + *C*_3_ ∙ *comorbidity*

^**e**^ The etiology coefficient has been back-transformed using the exponential function (*e*^*B*^) and thus values represent the ratio of EPO for each given etiology relative to the reference group

^f^ p<0.05

## Discussion

The present study is the largest conducted to date evaluating EPO levels for different etiologies of anemia in the elderly, and in particular AUE. Previous studies have suggested that the EPO response in AUE is inappropriately low however methodological limitations did not allow for definitive conclusions.[19] In the present study linear regression analysis showed that EPO levels in the CKD, ACD and AUE groups were lower by 48%, 46% and 27%, respectively, compared to IDA patients even after adjusting for hemoglobin, eGFR and comorbidities. Thus, our study confirms that compared to IDA, EPO levels are significantly lower in patients with AUE. This suggests that decreased erythropoietin production may play a key role in the pathogenesis of anemia of unknown etiology.

As expected, EPO levels were shown to be significantly lower in CKD, ACD and AUE compared to IDA. These results support the findings of our previous meta-analysis.[10] Furthermore, these results demonstrate that the inappropriately low EPO level in AUE is significant even after adjusting for confounders. Unsurprisingly, the group of patients with CKD had a significantly lower eGFR compared to IDA. AUE also had a significantly lower eGFR, suggesting that decreased renal function may partially account for the decreased EPO levels. Low EPO levels in CKD persist after adjustment for eGFR and suggest that the lower EPO response in CKD is not solely correlated with eGFR and that other factors are likely involved that determine the degree of anemia.

Our study shows that even after accounting for eGFR, EPO levels remain low in AUE. A possible mechanism for the decrease in EPO levels in this elderly population is the presence of a subclinical pro-inflammatory state. Inflammatory cytokines such as IL-1 and TNF alpha are postulated to play a role in development of anemia of chronic disease through inhibition of erythropoietin synthesis.[20] It is known that the elderly have increased levels of inflammatory cytokines, but it is unclear whether this is the result of the cardiovascular effects associated with aging or of the aging process itself.[21] Inflammatory markers such as C-reactive protein (CRP) and erythrocyte sedimentation rate (ESR) were collected as part of the study, but only a minority of patients underwent these tests. Therefore, retrospective analysis of the levels of inflammatory markers could not be reliably performed in our population.

Another potential contributing cause of AUE is the reduction of erythropoietic proliferative reserve that occurs with aging. It has been shown that the elderly have significantly lower numbers of bone marrow early erythroid-committed progenitors (BFU-E) compared to younger subjects, however comparison of anemic and non-anemic elderly populations demonstrates no difference in committed progenitor cell numbers or the effects of EPO on colony-forming unit erythroid (CFU-E) formation.[22] Moreover, our analysis demonstrates that erythropoietin levels are decreased in patients with anemia of unknown etiology, suggesting that the main cause is erythropoietin underproduction rather than decreased marrow reserve.

Erythropoietic pathways extrinsic to the EPO pathway may also play a role in the pathogenesis of AUE. It is known that both elderly men and women with lower testosterone levels are more likely to be anemic.[23] Testosterone, in addition to its potential effects on the EPO pathway, independently enhances proliferation of late BFU-E through stimulation of specific androgen nuclear receptors.[24] Another non-erythropoietin mediated pathway that may be implicated in AUE is activin-like kinase receptor 2 (ALK2) signaling. ALK2 plays an important role in the expression of hepcidin due to inflammatory cytokines and recent studies have focused on pharmacologically inhibiting this pathway.[25, 26] Future assessment of these alternative erythropoietic pathways may provide a more complete understanding of both anemia of chronic disease and anemia of unknown etiology.

A potential limitation of the present study is the use of IDA as a reference group rather than the use of an idealized control group that exhibits only acute anemia and that is otherwise normal. In order to compare the EPO levels in each etiology to expected values, we required a reference population of patients with a relatively normal EPO response to anemia. A reference population of anemic patients with a normal expected EPO response was not available retrospectively. Therefore we decided to use the group of patients with IDA for an approximation of the normal EPO response since patients are expected to demonstrate a more or less physiologic EPO response to anemia. However, both iron chelation and blockade of transferrin receptor-mediated iron uptake have been shown to stimulate EPO production, suggesting that iron deficiency itself may increase EPO levels.[27] There is also evidence that iron is required for degradation of HIF, suggesting that iron deficiency can increase levels of HIF and EPO levels.[28, 29] Data regarding the use of iron supplementation, which can decrease EPO levels, was not available at time of sampling.[30]

Our study has additional potential limitations. We cannot rule out the possibility of some degree of misclassification in the etiology of anemia. However, we believe this is less likely since we assigned anemia etiologies based on defined clinical criteria in order to maximize the objectivity of the groups and agreement between adjudicators was reasonable. We were also limited by the heterogeneity of patients within each etiologic group. Although AUE is classified as a single etiology, it is possible that a large amount of heterogeneity exists in the pathogenesis of the anemia in this patient population. Some of the patients in the AUE group had renal impairment that was not severe enough to meet the eGFR cutoff of 30 mL/min/1.73 m^2^. This GFR cutoff was chosen based on Mercadal et al., who demonstrated that the physiologic response to anemia is somewhat preserved in patients with GFRs greater than 30 mL/min/1.73 m^2^ and that there is no significant correlation between the EPO level and GFR above this cutoff.[14] Thus, in our study we focused on patients with severe CKD since patients with GFRs greater than 30 mL/min/1.73 m^2^ were unlikely to have CKD as the sole contributor to the anemia. Additionally, some patients in the AUE group had clinical features of MDS that were not confirmed with bone marrow findings, or a combination of borderline findings that did not meet diagnostic criteria by themselves but did suggest a multifactorial etiology—a limitation to all studies to date.

Finally, we recognize that including only patients in which EPO was measured introduces selection bias into the study but was difficult to avoid given our a priori interest in the role of EPO levels in this patient population.

In conclusion, our results suggest the EPO response is inadequate in elderly patients with AUE even after accounting for hemoglobin levels and renal function. This suggests that decreased EPO production or a blunted EPO response to anemia may play a role in the pathogenesis of AUE and that this may indeed constitute a distinct entity. Additional mechanisms may also be involved, including inflammation, reduced bone marrow reserve and erythropoietin-extrinsic pathways. Prospective studies are needed to confirm our findings and investigate potential mechanisms that may suggest future pharmacological interventions.

## Supporting Information

S1 File Raw experimental data. (XLSX)

S1 Table Baseline clinical characteristics of the study population by anemia etiology. Abbreviations: COPD, chronic obstructive pulmonary disease; MDS, myelodysplastic syndrome; SD, standard deviation. Note: The study identified 1 patient with vitamin B_12_ deficiency. There were no patients with folate deficiency.
(DOC)

S2 Table Baseline laboratory characteristics of the study population by anemia etiology. Abbreviations: CKD, chronic kidney disease; CRP, C-reactive protein; ESR, erythrocyte sedimentation rate; eGFR, estimated glomerular filtration rate; hsCRP, high-sensitivity C-reactive protein; MDS, myelodysplastic syndrome; SD, standard deviation; UIBC, unsaturated iron binding capacity. Note: The study identified 1 patient with vitamin B_12_ deficiency. There were no patients with folate deficiency.
(DOC)
