# Supplementary material for: Erythropoietin Levels in Elderly Patients with Anemia of Unknown Etiology
Source: PLoS One. 2016 Jun 16;11(6):e0157279. doi: 10.1371/journal.pone.0157279 (PMC4911007; doi:10.1371/journal.pone.0157279)
Supplement: S1 Table — Abbreviations: COPD, chronic obstructive pulmonary disease; MDS, myelodysplastic syndrome; SD, standard deviation. Note: The study identified 1 patient with vitamin B12 deficiency. There were no patients with folate deficiency. (DOC) [file pone.0157279.s002.doc]

|  | **Chronic kidney disease** | **Iron deficiency** | **Chronic disease** | **MDS** | **Suspected MDS** | **Unknown etiology** | **Other** | **Multi-factorial** | **All** |
| --- | --- | --- | --- | --- | --- | --- | --- | --- | --- |
| Number of patients | 25 | 59 | 31 | 180 | 19 | 117 | 118 | 20 | 570 |
| Male [no. (%)] | 12 (48.0) | 27 (45.8) | 18 (58.1) | 120 (66.7) | 12 (63.2) | 76 (65.0) | 68 (57.6) | 9 (45.0) | 342 (60.0) |
| Age [mean (SD)], y | 75.9 (8.9) | 75.3 (8.1) | 73.9 (7.7) | 75.7 (7.5) | 81.6 (7.3) | 76.2 (8.4) | 74.9 (8.4) | 76.3 (11.8) | 75.7 (8.2) |
| Weight [mean (SD)], kg | 76.2 (23.9) | 82.4 (22.9) | 69.2 (18.8) | 76.5 (14.8) | 78.7 (12.7) | 77.3 (20.9) | 70.3 (15.7) | 73.0 (17.1) | 75.5 (18.4) |
| Comorbidities |  |  |  |  |  |  |  |  |  |
| Type 1 diabetes mellitus [no. (%)] | 0 (0.0) | 1 (1.7) | 0 (0.0) | 1 (0.6) | 1 (5.3) | 0 (0.0) | 1 (0.8) | 0 (0.0) | 4 (0.7) |
| Type 2 diabetes mellitus [no. (%)] | 8 (32.0) | 25 (42.4) | 12 (38.7) | 39 (21.7) | 2 (10.5) | 54 (46.2) | 26 (22.0) | 7 (35.0) | 173 (30.4) |
| Chronic diabetes complications [no. (%)] | 6 (24.0) | 5 (8.5) | 3 (9.7) | 9 (5.0) | 1 (5.3) | 20 (17.1) | 7 (5.9) | 4 (20.0) | 55 (9.6) |
| Hypertension [no. (%)] | 18 (72.0) | 41 (69.5) | 17 (54.8) | 93 (51.7) | 10 (52.6) | 86 (73.5) | 64 (54.2) | 12 (60.0) | 341 (59.8) |
| History of stroke or TIA [no. (%)] | 6 (24.0) | 16 (27.1) | 6 (19.4) | 15 (8.3) | 5 (26.3) | 19 (16.2) | 14 (11.9) | 3 (15.0) | 84 (14.7) |
| Coronary artery disease [no. (%)] | 11 (44.0) | 16 (27.1) | 10 (32.3) | 65 (36.1) | 5 (26.3) | 54 (46.2) | 23 (19.5) | 8 (40.0) | 192 (33.7) |
| Congestive heart failure [no. (%)] | 6 (24.0) | 4 (6.8) | 4 (12.9) | 29 (16.1) | 3 (15.8) | 19 (16.2) | 10 (8.5) | 7 (35.0) | 82 (14.4) |
| Chronic kidney disease [no. (%)] | 23 (92.0) | 14 (23.7) | 13 (41.9) | 32 (17.8) | 2 (10.5) | 50 (42.7) | 24 (20.3) | 14 (70.0) | 172 (30.2) |
| Chronic liver disease [no. (%)] | 0 (0.0) | 9 (15.3) | 1 (3.2) | 9 (5.0) | 1 (5.3) | 6 (5.1) | 1 (0.8) | 3 (15.0) | 31 (5.4) |
| Liver disease is moderate/severe [no. (%)] | 0 (0.0) | 4 (6.8) | 0 (0.0) | 4 (2.2) | 0 (0.0) | 1 (0.9) | 0 (0.0) | 2 (10.0) | 11 (1.9) |
| Past solid malignancy [no. (%)] | 4 (16.0) | 16 (27.1) | 4 (12.9) | 25 (13.9) | 7 (36.8) | 12 (10.3) | 20 (16.9) | 2 (10.0) | 91 (16.0) |
| Active solid malignancy [no. (%)] | 0 (0.0) | 4 (6.8) | 3 (9.7) | 8 (4.4) | 0 (0.0) | 5 (4.3) | 3 (2.5) | 1 (5.0) | 24 (4.2) |
| Metastatic cancer [no. (%)] | 0 (0.0) | 1 (1.7) | 0 (0.0) | 1 (0.6) | 0 (0.0) | 0 (0.0) | 0 (0.0) | 0 (0.0) | 2 (0.4) |
| Past hematological cancer [no. (%)] | 0 (0.0) | 0 (0.0) | 0 (0.0) | 5 (2.8) | 0 (0.0) | 1 (0.9) | 1 (0.8) | 0 (0.0) | 7 (1.2) |
| Past myeloproliferative disorder [no. (%)] | 0 (0.0) | 0 (0.0) | 0 (0.0) | 1 (0.6) | 0 (0.0) | 0 (0.0) | 1 (0.8) | 0 (0.0) | 2 (0.4) |
| Active hematological cancer [no. (%)] | 4 (16.0) | 2 (3.4) | 0 (0.0) | 21 (11.7) | 2 (10.5) | 3 (2.6) | 53 (44.9) | 3 (15.0) | 88 (15.4) |
| Active myeloproliferative disorder [no. (%)] | 0 (0.0) | 4 (6.8) | 0 (0.0) | 2 (1.1) | 0 (0.0) | 0 (0.0) | 45 (38.1) | 1 (5.0) | 52 (9.1) |
| Vasculitis [no. (%)] | 1 (4.0) | 0 (0.0) | 3 (9.7) | 0 (0.0) | 0 (0.0) | 0 (0.0) | 0 (0.0) | 2 (10.0) | 6 (1.1) |
| Connective tissue disease [no. (%)] | 1 (4.0) | 1 (1.7) | 1 (3.2) | 2 (1.1) | 0 (0.0) | 0 (0.0) | 1 (0.8) | 0 (0.0) | 6 (1.1) |
| Autoimmune disorder [no. (%)] | 3 (12.0) | 3 (5.1) | 4 (12.9) | 6 (3.3) | 0 (0.0) | 2 (1.7) | 1 (0.8) | 0 (0.0) | 19 (3.3) |
| Rheumatoid arthritis [no. (%)] | 0 (0.0) | 0 (0.0) | 13 (41.9) | 11 (6.1) | 0 (0.0) | 3 (2.6) | 7 (5.9) | 2 (10.0) | 36 (6.3) |
| Other rheumatologic condition [no. (%)] | 0 (0.0) | 2 (3.4) | 7 (22.6) | 1 (0.6) | 0 (0.0) | 0 (0.0) | 0 (0.0) | 1 (5.0) | 11 (1.9) |
| Osteoarthritis [no. (%)] | 5 (20.0) | 15 (25.4) | 6 (19.4) | 35 (19.4) | 7 (36.8) | 29 (24.8) | 22 (18.6) | 4 (20.0) | 123 (21.6) |
| Dementia [no. (%)] | 2 (8.0) | 3 (5.1) | 2 (6.5) | 3 (1.7) | 1 (5.3) | 6 (5.1) | 2 (1.7) | 1 (5.0) | 20 (3.5) |
| COPD [no. (%)] | 5 (20.0) | 9 (15.3) | 3 (9.7) | 16 (8.9) | 2 (10.5) | 14 (12.0) | 14 (11.9) | 2 (10.0) | 65 (11.4) |
| Comorbidity Index [mean (SD)] | 2.5 (1.5) | 1.5 (1.6) | 2.0 (1.4) | 1.3 (1.5) | 1.0 (1.2) | 1.4 (1.4) | 1.6 (1.3) | 3.0 (1.9) | 1.5 (1.5) |
