# Supplementary material for: Erythropoietin Levels in Elderly Patients with Anemia of Unknown Etiology
Source: PLoS One. 2016 Jun 16;11(6):e0157279. doi: 10.1371/journal.pone.0157279 (PMC4911007; doi:10.1371/journal.pone.0157279)
Supplement: S2 Table — Abbreviations: CKD, chronic kidney disease; CRP, C-reactive protein; ESR, erythrocyte sedimentation rate; eGFR, estimated glomerular filtration rate; hsCRP, high-sensitivity C-reactive protein; MDS, myelodysplastic syndrome; SD, standard deviation; UIBC, unsaturated iron binding capacity. Note: The study identified 1 patient with vitamin B12 deficiency. There were no patients with folate deficiency. (DOC) [file pone.0157279.s003.doc]

|  | **Chronic kidney disease** | **Iron deficiency** | **Chronic disease** | **MDS** | **Suspected MDS** | **Unknown etiology** | **Other** | **Multi-factorial** | **All** |
| --- | --- | --- | --- | --- | --- | --- | --- | --- | --- |
| Number of patients | 25 | 59 | 31 | 180 | 19 | 117 | 118 | 20 | 570 |
| Erythropoietin [mean (SD)], IU/L | 25.1 (40.4) | 102.4 (148.6) | 26.0 (19.6) | 287.8 (768.0) | 148.1 (230.5) | 39.1 (82.2) | 271.4 (552.5) | 106.1 (231.8) | 176.9 (517.9) |
| Hemoglobin [mean (SD)] g/L | 96.6 (13.7) | 95.6 (15.8) | 99.9 (13.3) | 91.1 (14.5) | 97.0 (15.6) | 105.6 (13.2) | 92.7 (16.9) | 93.2 (14.0) | 95.9 (15.7) |
| Mean corpuscular volume [mean (SD)], fL | 96.6 (7.8) | 83.9 (7.3) | 93.8 (9.8) | 97.8 (9.8) | 97.5 (9.3) | 93.5 (8.2) | 95.9 (11.9) | 90.9 (7.5) | 94.6 (10.4) |
| Reticulocytes [mean (SD)], x10^9^/L | 53.9 (40.6) | 67.4 (25.6) | 50.2 (17.1) | 82.1 (99.9) | 43.5 (27.3) | 45.2 (18.2) | 80.6 (96.7) | 50.3 (23.9) | 66.3 (72.5) |
| Leukocytes [mean (SD)], x10^9^/L | 7.1 (2.9) | 7.3 (4.5) | 7.5 (3.3) | 6.2 (6.5) | 5.4 (3.7) | 6.6 (2.5) | 12.4 (19.1) | 9.1 (8.3) | 7.9 (10.0) |
| Thrombocytes [mean (SD)], x10^9^/L | 234 (126) | 284 (224) | 252 (137) | 162 (131) | 157 (146) | 211 (108) | 288 (322) | 240 (132) | 221 (200) |
| Neutrophils [mean (SD), x10^9^/L | 5.1 (2.6) | 4.9 (2.5) | 5.7 (3.0) | 3.3 (3.6) | 3.2 (2.4) | 4.5 (2.2) | 5.6 (8.1) | 6.1 (7.2) | 4.5 (4.8) |
| Creatinine [mean (SD)], µmol/L | 271 (150) | 98 (39) | 110 (39) | 102 (42) | 95 (23) | 115 (34) | 110 (78) | 167 (83) | 116 (69) |
| CKD-EPI formula |  |  |  |  |  |  |  |  |  |
| eGFR [mean (SD)], mL/min/1.73 m^2^ | 20.2 (7.4) | 63.3 (23.9) | 57.4 (20.6) | 63.7 (21.9) | 61.6 (17.0) | 53.6 (19.0) | 62.7 (23.8) | 37.8 (22.9) | 58.1 (23.5) |
| Severe CKD, eGFR<30 [no. (%)] | 25 (100.0) | 5 (8.5) | 1 (3.2) | 14 (7.8) | 0 (0.0) | 0 (0.0) | 6 (5.1) | 11 (55.0) | 62 (10.9) |
| Moderate CKD, 30<eGFR<60 [no. (%)] | 0 (0.0) | 24 (40.7) | 15 (48.4) | 65 (36.1) | 9 (47.4) | 78 (66.7) | 46 (39.0) | 6 (30.0) | 244 (42.8) |
| No/mild CKD, eGFR>60 [no. (%)] | 0 (0.0) | 30 (50.8) | 15 (48.4) | 99 (55.0) | 10 (52.6) | 38 (32.5) | 58 (49.2) | 3 (15.0) | 173 (30.4) |
| Ferritin [mean (SD)], ug/L | 322 (500) | 32 (48) | 507 (812) | 696 (749) | 747 (1,273) | 270 (363) | 682 (969) | 286 (417) | 479 (734) |
| Iron [mean (SD)], ug/L | 12.5 (5.5) | 5.5 (3.1) | 9.7 (5.5) | 18.4 (12.0) | 12.5 (3.4) | 13.4 (5.6) | 18.5 (12.0) | 9.1 (3.6) | 13.9 (9.5) |
| UIBC [mean (SD)], µmol/L | 31.2 (9.4) | 59.8 (10.9) | 29.3 (9.4) | 28.0 (17.0) | 29.5 (8.9) | 37.1 (13.3) | 20.5 (13.4) | 49.0 (13.7) | 34.1 (17.6) |
| Transferrin saturation [mean (SD)], % | 29.5 (13.8) | 8.7 (5.3) | 25.5 (18.1) | 37.4 (23.8) | 31.0 (10.6) | 27.6 (12.8) | 44.4 (28.4) | 15.4 (4.5) | 29.5 (21.2) |
| Serum B12 [mean (SD)], pmol/L | 320 (160) | 499 (338) | 514 (359) | 534 (355) | 415 (159) | 509 (352) | 524 (368) | 466 (415) | 503 (347) |
| Erythrocyte folate [mean (SD)], nmol/L | 2,273 (1,063) | 1,864 (723) | 2,016 (807) | 2,126 (847) | 2,060 (1,152) | 1,908 (687) | 2,054 (950) | 1,936 (647) | 2,026 (836) |
| Serum folate [mean (SD)], nmol/L | 26.9 (7.7) | 32.6 (23.9) | 40.6 (17.1) | 33.7 (15.7) | 32.1 (12.9) | 32.5 (14.8) | 32.0 (16.6) | 28.4 (11.2) | 32.6 (15.2) |
| CRP [mean (SD)], mg/L | 10.2 (8.3) | 35.9 (59.1) | 12.3 (14.9) | 42.1 (55.3) | 6.6 (2.0) | 32.1 (81.3) | 12.1 (10.7) | - | 28.6 (52.8) |
| hsCRP [mean (SD)], mg/L | 4.8 (2.9) | - | - | 129.5 (217.0) | - | - | 31.4 (46.2) | - | 60.1 (110.1) |
| ESR [mean (SD)], mm/hr | 67.7 (42.0) | 48.0 (18.7) | 67.1 (38.4) | 46.4 (31.8) | - | 43.9 (35.8) | 57.8 (31.6) | 73.5 (30.6) | 54.6 (34.7) |
